# Effects of repetitive transcranial magnetic stimulation therapy on weight and lipid metabolism in patients with treatment‐resistant depression: A preliminary single‐center retrospective cohort study

**DOI:** 10.1002/npr2.12494

**Published:** 2024-11-09

**Authors:** Ami Nakazawa, Yuki Matsuda, Ryuichi Yamazaki, Nanase Taruishi, Shinsuke Kito

**Affiliations:** ^1^ Department of Psychiatry Jikei University School of Medicine Minato‐ku Tokyo Japan; ^2^ Department of Psychiatry, National Center Hospital National Center of Neurology and Psychiatry Minato‐ku Tokyo Japan

**Keywords:** lipid metabolism, repetitive transcranial magnetic stimulation, treatment‐resistant depression, weight

## Abstract

**Aim:**

This study aimed to elucidate the effects of repetitive transcranial magnetic stimulation (rTMS) on weight, body mass index (BMI), and lipid metabolism in patients with treatment‐resistant depression (TRD).

**Methods:**

This retrospective observational study included patients with TRD who received rTMS treatment at the Jikei University Hospital from September 2018 to August 2021. The patients were diagnosed based on the DSM‐5 and ICD‐10 criteria and treated using the NeuroStar TMS System. For 3–6 weeks, 10‐Hz rTMS was administered to the left dorsolateral prefrontal cortex at 120% motor threshold. The primary outcomes were changes in weight and BMI, whereas the secondary outcomes included changes in total, high‐density lipoprotein (HDL), and low‐density lipoprotein (LDL) cholesterol levels, thyroid function indicators, as well as HAMD‐17, HAMD‐24, and Montgomery–Åsberg Depression Rating Scale (MADRS) scores. Statistical analysis was conducted using paired *t*‐tests and repeated measures ANOVA.

**Results:**

Among the 34 patients (20 men and 14 women) included, no significant changes were observed in weight or BMI after rTMS treatment (average weight reduction: −0.50 kg, 95% CI: −0.14 to 0.56, *p* = 0.24; average BMI reduction: −0.21, 95% CI: −0.10 to 0.61, *p* = 0.15). However, significant reductions in total, HDL, and LDL cholesterol levels and FT4 were observed. Furthermore, the HAMD‐17, HAMD‐24, and MADRS scores significantly increased post‐treatment.

**Conclusion:**

rTMS treatment did not affect weight or BMI in patients with TRD but is believed to improve lipid metabolism.

## Abstract 2

This study investigated changes in weight, BMI, and blood test results before and after repetitive transcranial magnetic stimulation (rTMS) treatment in TRD patients.

## Graphical abstract

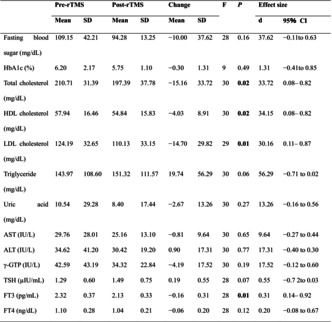

## 1 INTRODUCTION

Major depressive disorder (MDD) is one of the most prevalent mental health conditions worldwide, affecting approximately 280 million people. In 2019, it ranked 13th in terms of disability‐adjusted life years,^1^ thus representing a significant public health challenge. MDD leads to impaired social functioning, increased unemployment rate, a higher risk of physical illness, and increased mortality rate due to physical diseases and suicide.^2^ According to major guidelines, antidepressants are the first‐line treatment for moderate‐to‐severe depression.^3^ Treatment‐resistant depression (TRD), defined as MDD not remitting after adequate doses and durations of at least two antidepressants,^4^ is estimated to affect 30.9% of patients with MDD in the United States, leading to increased healthcare costs compared with non‐TRD patients.^5^

Repetitive transcranial magnetic stimulation (rTMS) is a nonpharmacological treatment for TRD that employs the principle of electromagnetic induction, as described by Faraday's law.^6^ It can enhance or inhibit cortical excitability depending on the stimulation frequency.^6^ Multiple randomized controlled trials have reported its efficacy in TRD, demonstrating significant improvements in depression symptoms and higher response and remission rates compared with sham stimulation.^7^, ^8^

Patients with MDD are susceptible to obesity, dyslipidemia, hyperglycemia, and hypertension due to decreased activity, smoking habit, alcohol use, sleep disorders, and unhealthy eating habits.^9^ Over 50.8% of patients with TRD have a body mass index (BMI) over 25, and 38% meet the criteria for metabolic syndrome; these rates are higher than those observed in other mental disorders.^10^ Augmentation strategies are effective in treating TRD.^11^, ^12^ However, the use of antidepressants and antipsychotics can lead to weight gain.^13^, ^14^, ^15^

Although rTMS has shown efficacy under various psychiatric and neurological conditions, including effects on obesity,^16^, ^17^, ^18^ its impact on weight and lipid metabolism in patients with TRD, who are already at a high risk for weight gain and metabolic syndrome, has not yet been explored. Therefore, this study examined the changes in weight, BMI, and blood test results before and after rTMS treatment in TRD patients treated at the Jikei University Hospital from September 1, 2018, to August 31, 2021.

## 2 METHODS

### 2.1 Study design and setting

This retrospective observational study utilized patient data obtained from “The effects of rTMS for cognitive function of depression: prospective observational study” (UMIN: 000044836). Patients with TRD who received rTMS treatment at Jikei University Hospital from September 1, 2018, to August 31, 2021, were included. This study was conducted in accordance with the tenets of the 2013 revision of the Declaration of Helsinki and approved by the Ethics Committee of Jikei University (approval no. 30–154[9175] on December 13, 2021). The study's objectives and details were posted in an opt‐out document on the website of Jikei University Hospital to fully inform patients of the study details and provide an opportunity to refuse consent to the use of their data.

### 2.2 Study participants

The participants were diagnosed by two psychiatrists according to the Diagnostic and Statistical Manual of Mental Disorders, Fifth Edition, and the International Statistical Classification of Diseases and Related Health Problems. Patients aged 18 or above who had received optimal doses of at least one antidepressant but had not achieved remission were included. Patients were excluded if they were below 18 years old, had previously received rTMS for the same depressive episode, had organic diseases, did not meet the diagnostic criteria for a moderate depressive episode, exhibited anxiety depression symptoms, had severe episodes with psychotic symptoms, had imminent suicidal thoughts or somatic syndrome, had residual affective disorders caused by psychoactive substances, or had contraindications for rTMS such as metal in the stimulation area or a pacemaker. Outpatients whose weight changes were not monitored during the treatment period, who did not have their weight measured before and after the treatment, and who consumed a calorie‐restricted diet during hospitalization were also excluded.

### 2.3 TMS procedures

The rTMS treatment (10 Hz) was administered using the NeuroStar TMS System (Neuronetics, Inc., Malvern, PA, USA), targeting the left dorsolateral prefrontal cortex (LDLPFC) at 120% motor threshold (MT) with 4‐s stimulation and 26‐s intervals, totaling 3000 pulses per day. The treatment consisted of one session per day, 5 days a week, for 3–6 weeks. The NeuroStar TMS System employs an iron‐core coil, which provides high focality at the stimulation site.^19^, ^20^ The standard rTMS therapy stimulation intensity for MDD is 120% of the MT.^7^, ^8^ This protocol was followed in the present study. Remission was defined as a HAMD‐17 score of 7 or lower. If patients met the remission criteria after 3 weeks, the rTMS treatment was discontinued or tapered. If an improvement of less than 20% from baseline was attained and the remission criteria were not met, the treatment was discontinued. If a response was obtained but remission was not achieved, the treatment was continued up to 6 weeks or a maximum of 30 sessions. The MT position was identified using an automated pulse emission mode by targeting the right abductor pollicis brevis, searching for thumb contraction. The position of the treatment coil that consistently induced thumb contraction at the lowest MT level was defined as the MT position. The stimulation intensity was set at 120% MT, determined using the MT Assist program (MT Assist®, Neuronetics, Inc., Malvern, PA, USA). The treatment coil was positioned 5.5 cm anterior along the midsagittal line from the MT position. If 120% MT was difficult to achieve due to adverse events, treatment was administered at 100%–120% MT. The dosage of psychotropic drugs the patients were already taking was maintained during the rTMS treatment. Antihypertensive, antidiabetic, and lipid‐lowering medications were also continued at the same doses.

### 2.4 Hospital diet

Male patients aged 18–74 years consumed a hospital diet of 1900 kcal/day, whereas male patients over 75 years and female patients over 18 years consumed 1600 kcal/day. Patients with a history of lifestyle diseases were provided with an energy‐adjusted diet. Such a diet ranged from 960 to 2000 kcal, selected by the attending physician based on the patient's height and standard weight. The daily calorie intake was calculated as standard weight × 22 kcal. The closest calorie intake to this calculation was selected. No restrictions were placed on snacks or drinking water outside the hospital‐provided meals.

### 2.5 Evaluation items

The primary evaluation items were changes in weight and BMI before and after rTMS treatment, whereas the secondary evaluation items included changes in the levels of blood glucose, HbA1c, total cholesterol, HDL cholesterol, LDL cholesterol, triglyceride (TG), AST, alanine transaminase (ALT), γ‐GTP, thyroid‐stimulating hormone (TSH), free T4 (FT4), and free T3 (FT3). Considering the impact on glucose metabolism, tests were conducted after fasting or before breakfast. Depression symptoms were evaluated by a psychiatrist using MADRS, HAMD‐17, and HAMD‐24 at baseline upon hospital admission and then weekly after treatment initiation. Adverse events were evaluated at each treatment session using a side‐effect checklist.

### 2.6 Statistical analysis

Changes in weight, BMI, blood tests, and depression rating scales before and after the treatment were analyzed using paired *t*‐tests. Changes in weight and BMI from the start of treatment to week 6 were analyzed using repeated measures analysis of variance. The tests were two‐tailed, and *p* < 0.05 was considered to indicate statistical significance. As this study was exploratory, no imputation for missing values or correction for multiple testing was performed. To investigate the effects of rTMS on weight and lipid metabolism, we divided our patients into two BMI subgroups, with a BMI of 22 as the cutoff. Thus, there was a low BMI group (BMI <22) and a high BMI group (BMI ≥22). This classification of participants was selected because of the small sample size, which prevented more granular subgroup analyses. Statistical analysis was conducted using SPSS version 28.0 (IBM Corp., Armonk, NY, USA) software.

## 3 RESULTS

### 3.1 Patients

**FIGURE 1:**
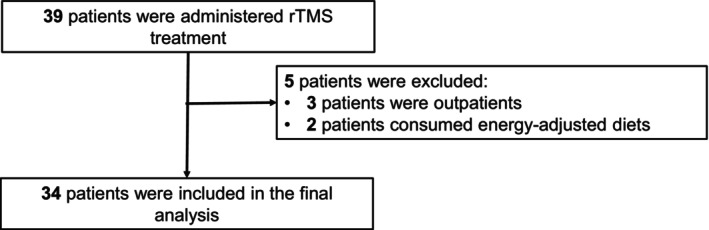
Patient selection flowchart. This flowchart illustrates the patient selection process for inclusion in an analysis of rTMS treatment. Initially, 39 patients received rTMS treatment. A total of five patients were excluded based on specific criteria: Three were outpatients and two followed energy‐adjusted diets. Finally, 34 patients were included in the analysis.

The patient selection flowchart is presented in Figure 1. During the study period, the rTMS treatment was administered to 39 patients. After excluding three outpatients and two patients who consumed energy‐adjusted meals, 34 (20 men and 14 women) were finally included in the analysis. Table 1 presents the characteristics of these patients. Their average age was 48.76 years; average height, 167.68 cm; weight at admission, 67.60 kg; and BMI, 24.09, indicating a normal weight range. The history of lifestyle diseases included hypertension (*n* = 7, 20.6%), diabetes (*n* = 5, 14.7%), hyperlipidemia (*n* = 8, 23.5%), and hyperuricemia (*n* = 2, 5.9%). The average number of rTMS sessions was 26.21, the average duration of rTMS treatment was 39.12 days, and the average hospital stay was 55.15 days. The baseline HAMD‐17 score was 17.57, suggesting moderate depression. The psychotropic medications used included selective serotonin reuptake inhibitors (*n* = 5, 14.7%), serotonin–noradrenaline reuptake inhibitors (*n* = 12, 35.3%), noradrenaline and specific serotonergic antidepressants (*n* = 4, 11.8%), tricyclic antidepressants (*n* = 5, 14.7%), tetracyclic antidepressants (*n* = 3, 8.8%), serotonin antagonist and reuptake inhibitors (*n* = 3, 8.8%), mood stabilizers (*n* = 10, 29.4%), typical antipsychotics (*n* = 2, 5.9%), and atypical antipsychotics (*n* = 6, 17.6%). There were no changes in medication regimens during the rTMS treatment period.

**TABLE 1:** Baseline demographics of the participants.

Characteristic or measure		
	Mean	SD
Age (years)	48.76	13.16
Onset age (years)	36.09	15.17
Duration of current depressive episode (months)	36.82	42.97
Education (years)	15.26	2.14
HAMD‐17	17.57	3.93
HAMD‐24	22.28	3.90
MADRS	23.58	6.30
Number of rTMS treatment (sessions)	26.21	6.44
Duration of treatment (days)	39.12	9.76
Hospitalization (days)	55.15	30.13
Height (cm)	167.68	7.85
Body weight (kg)	67.60	15.43
BMI (kg/m^2^)	24.09	5.00

	*n*	%
Female	14	41.2
Comorbidity		
Hypertension	7	20.6
Diabetes mellitus	5	14.7
Hyperlipidemia	8	23.5
Hyperuricemia	2	5.9
Medication		
SSRI	5	14.7
SNRI	12	35.3
NaSSA	4	11.8
TCA	5	14.7
TeCA	3	8.8
SARI	3	8.8
Mood stabilizer	10	29.4
FGA	2	5.9
SGA	6	17.6

Abbreviations: BMI, body mass index; FGA, first‐generation antipsychotic; HAMD, Hamilton Depression Rating Scale; MADRS, Montgomery–Åsberg Depression Rating Scale; *n*, number of patients; NaSSA, noradrenergic and specific serotonergic antidepressant; rTMS, repetitive transcranial magnetic stimulation; SARI, serotonin antagonist and reuptake inhibitor; SD, standard deviation; SGA, second‐generation antipsychotic; SNRI, serotonin–noradrenaline reuptake inhibitor; SSRI, selective serotonin reuptake inhibitor; TCA, tricyclic antidepressant; TeCA, tetracyclic antidepressant.

### 3.2 Changes in weight and BMI

The changes in weight and BMI before and after the rTMS treatment are shown in Table 2. The average weight decreased from 67.60 to 67.10 kg, indicating a reduction of 0.50 kg, but this change was not statistically significant (95% confidence interval [CI]: −0.14 to 0.56, *p* = 0.24). Similarly, the average BMI decreased from 24.09 to 23.89, indicating a reduction of 0.21, but this change was also not statistically significant (95% CI: −0.10 to 0.61, *p* = 0.15).

**TABLE 2:** Changes in body weight and BMI.

	Pre‐rTMS		Post‐rTMS		Change		*F*	*p*	Effect size	
	Mean	SD	Mean	SD	Mean	SD			*d*	95% CI
Body weight (kg)	67.60	15.43	67.10	14.61	−0.50	2.34	31	0.24	2.34	−0.14 to 0.56
BMI (kg/m^2^)	24.09	5.00	23.89	4.79	−0.21	0.80	31	0.15	0.80	−0.10 to 0.61

Abbreviations: 95% CI, 95% confidence interval; BMI, body mass index; *d*, Cohen's *d*; rTMS, repetitive transcranial magnetic stimulation; SD, standard deviation.

### 3.3 Changes in blood tests

The changes in blood test results before and after the rTMS treatment are shown in Table 3. Significant decreases were observed in the levels of total cholesterol (210.71 vs. 197.39 mg/dL, *p* = 0.02), HDL cholesterol (57.94 vs. 54.84 mg/dL, *p* = 0.02), and LDL cholesterol (197.39 vs. 110.13 mg/dL, *p* = 0.02) after the treatment. Furthermore, there was an increasing trend in the TSH levels (1.29 vs. 1.49 μIU/mL, *p* = 0.07) and a significant decrease in the FT3 levels (2.32 vs. 2.13 pg/mL, *p* = 0.01).

**TABLE 3:** Changes in laboratory data.

	Pre‐rTMS		Post‐rTMS		Change		F	*p*	Effect size	
	Mean	SD	Mean	SD	Mean	SD			d	95% CI
Fasting blood sugar (mg/dL)	109.15	42.21	94.28	13.25	−10.00	37.62	28	0.16	37.62	−0.11 to 0.63
HbA1c (%)	6.20	2.17	5.75	1.10	−0.30	1.31	9	0.49	1.31	−0.41 to 0.85
Total cholesterol (mg/dL)	210.71	31.39	197.39	37.78	−15.16	33.72	30	**0.02**	33.72	0.08 to 0.82
HDL cholesterol (mg/dL)	57.94	16.46	54.84	15.83	−4.03	8.91	30	**0.02**	34.15	0.08 to 0.82
LDL cholesterol (mg/dL)	124.19	32.65	110.13	33.15	−14.70	29.82	29	**0.01**	30.16	0.11 to 0.87
Triglyceride (mg/dL)	143.97	108.60	151.32	111.57	19.74	56.29	30	0.06	56.29	−0.71 to 0.02
Uric acid (mg/dL)	10.54	29.28	8.40	17.44	−2.67	13.26	30	0.27	13.26	−0.16 to 0.56
AST (IU/L)	29.76	28.01	25.16	13.10	−0.81	9.64	30	0.65	9.64	−0.27 to 0.44
ALT (IU/L)	34.62	41.20	30.42	19.20	0.90	17.31	30	0.77	17.31	−0.40 to 0.30
γ‐GTP (IU/L)	42.59	43.19	34.32	22.84	−4.19	17.52	30	0.19	17.52	−0.12 to 0.60
TSH (μIU/mL)	1.29	0.60	1.49	0.75	0.19	0.55	28	0.07	0.55	−0.7 2 to 0.03
FT3 (pg/mL)	2.32	0.37	2.13	0.33	−0.16	0.31	28	**0.01**	0.31	0.14 to 0.92
FT4 (ng/dL)	1.10	0.28	1.04	0.21	−0.06	0.20	28	0.12	0.20	−0.08 to 0.67

*Note*: Bold values indicates significant differences.

Abbreviations: 95% CI, 95% confidence interval; ALT, alanine aminotransferase; AST, aspartate aminotransferase; d, Cohen's d; FT3, free triiodothyronine; FT4, free thyroxine; HbA1c, hemoglobin A1c; HDL, high‐density lipoprotein; LDL, low‐density lipoprotein; rTMS, repetitive transcranial magnetic stimulation; SD, standard deviation; TSH, thyroid‐stimulating hormone; γ‐GTP, γ‐glutamyl transpeptidase.

### 3.4 Changes in depression symptoms

The changes in depression symptoms are shown in Table 4. There were significant improvements in the HAMD‐17 (17.57 vs. 6.53, *p* = 0.01), HAMD‐24 (22.28 vs. 7.79, *p* = 0.01), and MADRS (23.58 vs. 8.36, *p* = 0.01) scores after the treatment.

**TABLE 4:** Changes in clinical assessment scores.

	Pre‐rTMS		Post‐rTMS		Change		*F*	*p*	Effect size		Response (*n*, %)^a^	Remission (*n*, %)^b^
	Mean	SD	Mean	SD	Mean	SD			*d*	95% CI		
HAMD‐17	17.57	3.93	6.53	4.84	−11.03	5.63	29	**<0.01**	5.63	1.34–2.57	30, 88.24	20, 58.82
HAMD‐24	22.28	3.90	7.79	5.00	−14.48	5.13	28	**<0.01**	5.13	2.00–3.64		
MADRS	23.58	6.30	8.36	7.75	−15.21	7.54	32	**<0.01**	7.54	1.41–2.61		

*Note*: Bold values indicates significant differences.

Abbreviations: 95% CI, 95% confidence interval; *d*, Cohen's *d*; HAMD, Hamilton Depression Rating Scale; MADRS: Montgomery–Åsberg Depression Rating Scale; *n*, number of patients; rTMS, repetitive transcranial magnetic stimulation; SD, standard deviation.

^a^ Response was defined as a reduction ≥50% in the HAMD‐17 score from baseline.

^b^ Remission was defined as a score ≤7 on the HAMD‐17 score.

### 3.5 Subgroup analysis

Analysis of the two BMI subgroups (low BMI <22 and high BMI ≥22) showed no significant changes in weight or BMI in either group (Table S1). However, nonsignificant trends were observed in both groups toward reductions in total cholesterol, LDL cholesterol, and HDL cholesterol, with the high BMI group showing more substantial changes in weight, BMI, and lipid metabolism.

## 4 DISCUSSION

This study explored the effects of rTMS treatment on weight, BMI, and blood tests in patients with TRD. Although rTMS did not significantly change weight or BMI, it significantly altered lipid metabolism and thyroid function, indicating that rTMS may impact physical metabolic functions.

Previous studies have reported that rTMS affects weight reduction in patients with obesity. For example, in a randomized controlled trial (RCT) of 29 patients with obesity receiving 10‐Hz stimulation at 110% MT to the LDLPFC for 2000 pulses per day over 4 days, the treatment group exhibited a significant weight reduction after 2 weeks compared with the control group (−1.35 ± 2.31 kg vs. 0.45 ± 1.28 kg, *p* < 0.002).^17^ Another RCT involving 21 patients with obesity under the same stimulation conditions over 8 days exhibited significant weight reduction after 4 weeks (−2.75 ± 2.37 kg vs. 0.38 ± 1.0 kg, *p* < 0.01), along with improvements in BMI, appetite, hunger, daily calorie intake, fasting blood sugar, and insulin resistance.^18^ Furthermore, a meta‐analysis of brain stimulation treatments for obesity showed a significant reduction in BMI in the group receiving high‐frequency rTMS targeting the LDLPFC compared with the control group (SMD: −1.34, 95% CI: −2.24 to −0.43).^21^

However, unlike previous studies that focused on patients with obesity with a BMI over 25 and often above 30 and who were not hospitalized and chose their meals, our study did not observe significant changes in weight or BMI. Such a discrepancy could be attributed to differences in the study populations and conditions. In the present study, the patients had an average BMI of 24 and were hospitalized, which led to lower energy expenditure and a consistent calorie intake due to fixed hospital meals. Moreover, a high proportion of patients were taking antidepressants and antipsychotics, which could contribute to weight gain.

It is established that patients with MDD exhibit higher total cholesterol levels than healthy individuals.^22^ Sonawalla et al. found that MDD patients with total cholesterol levels exceeding 200 mg/dL respond less effectively to pharmacotherapy.^23^ Similarly, Papakostas et al. compared blood tests between MDD and TRD patients and found a tendency for higher total cholesterol levels in the TRD group (202.0 vs. 196.8 mg/dL, *p* = 0.12), indicating that total cholesterol levels above 200 mg/dL are correlated with poor prognosis.^24^ Our study found an average total cholesterol level of 210.7 mg/dL among the patients, consistent with previous findings indicating that levels above 200 mg/dL predict poor outcomes.^24^ Consequently, this suggests poor prognosis and diminished pharmacotherapy responsiveness in this cohort.

A few studies have investigated the impact of rTMS on lipid metabolism. Kim et al. reported a trend toward reduced TG and LDL cholesterol levels after 4 weeks of rTMS in patients with obesity.^17^ In addition, Ren et al. observed significant reductions in total cholesterol and TG levels in healthy elderly men after stimulation of the right DLPFC.^25^ These findings support the potential of rTMS to affect lipid metabolism. The changes in lipid metabolism observed in this study could be attributed to the activation of the hypothalamic–pituitary–thyroid (HPT) axis by rTMS, as indicated by the increase in TSH levels and decreases in total, HDL, and LDL cholesterol levels. Several studies have reported that rTMS treatment affects the HPT axis. Szuba et al. conducted a study involving 14 MDD patients, applying 10‐Hz stimulation to the LDLPFC at 100% MT, with 1000 stimuli per day. They found that the TSH levels immediately after the rTMS treatment were significantly higher than those after a sham stimulation.^26^ Similarly, Kito et al. applied 1‐Hz stimulation to the right DLPFC of patients with TRD, with 3600 stimuli per day, and found a notable increase in serum TSH levels.^27^ In the present study, the TSH levels exhibited an increasing trend, whereas the total, HDL, and LDL cholesterol levels markedly decreased. These findings are consistent with those of previous research, suggesting that rTMS treatment affects lipid metabolism through the HPT axis. Furthermore, rTMS affects the autonomic nervous system. A previous study suggested that the therapeutic effects of rTMS are linked to changes in autonomic function.^28^ rTMS of the prefrontal cortex may influence the hypothalamus and brainstem, which are crucial in autonomic regulation, potentially leading to alterations in autonomic function. However, the precise mechanisms underlying these effects remain unclear.

In addition to the increasing trend observed in TG levels, previous studies have shown a negative correlation between physical activity and TG levels,^29^ and both TG and HDL cholesterol levels are particularly sensitive to changes in physical activity.^30^ Given that this study was conducted in an inpatient setting, it is possible that reduced physical activity contributed to the increase in TG levels and the decrease in HDL cholesterol levels.

This study has several limitations. First, the small sample size and single‐center design limit the generalizability of the results. Second, the hospital setting and diet could have influenced the outcomes because hospitalized patients typically have lower activity levels, potentially reducing calorie expenditure and affecting weight changes. Furthermore, the prescribed hospital diet might have contributed to improved lipid metabolism. Third, variations in medication treatments among the study population were not considered. Fourth, our study demonstrated higher response and remission rates than the NeuroStar TMS System registry trials conducted in the United States.^31^, ^32^ This finding could be attributed to the significant expertise in rTMS treatment at Jikei University Hospital, a high‐volume center in Japan. Moreover, unlike previous studies conducted in outpatient settings, our study was conducted in an inpatient setting. Hospitalization may have enhanced the therapeutic effects of rTMS through factors such as structured daily routines, stress reduction, and support from medical staff, which we recognize as influences on the therapeutic effects and limitations of our study.

This study is the first to report on the effect of rTMS treatment on lipid metabolism in patients with TRD. In general, patients with TRD are at a high risk of weight gain and metabolic syndrome, and augmentation therapies with antidepressants and antipsychotics could further increase such risks. Our findings suggest that rTMS not only improves depressive symptoms but also enhances lipid metabolism without increasing weight, offering a potential treatment option for patients with TRD, particularly those at risk for obesity and lipid metabolism disorders. A subgroup analysis of participants with high and low BMIs showed that rTMS exerted a more pronounced effect on lipid metabolism in the high BMI group (BMI ≥22). However, changes in weight and BMI did not reach statistical significance in either group. Future studies should include a control group of TRD patients not receiving rTMS during hospitalization in a prospective comparative trial.

This study investigated changes in weight, BMI, and blood tests in TRD patients before and after the rTMS treatment. The findings indicate that rTMS treatment does not affect weight or BMI in patients with TRD and may enhance lipid metabolism.

## AUTHOR CONTRIBUTIONS

AN and YM were involved in the study conception and design, conducted the statistical analysis, and interpreted the data. RY and NT performed the data acquisition. All the authors contributed to the manuscript, and SK supervised the study.

## FUNDING INFORMATION

This work was supported by the Jikei University School of Medicine Women's Researcher Career Support System.

## CONFLICT OF INTEREST STATEMENT

AN has no conflicts of interest to declare. YM has received speaker's honoraria from Otsuka Pharmaceutical Co., Ltd.; Sumitomo Pharma Co., Ltd.; Takeda Pharmaceutical Co., Ltd.; Teijin Pharma Ltd.; Lundbeck Japan K.K.; and Viatris Inc. RY has received a speaker's honoraria from Inter Reha Co., Ltd. NT has no conflicts of interest to declare. SK has received speaker's honoraria from Inter Reha Co., Ltd.; Lundbeck Japan K.K.; Sumitomo Pharma Co., Ltd.; Otsuka Pharmaceutical Co., Ltd.; Takeda Pharmaceutical Co., Ltd.; Teijin Pharma Ltd.; and Viatris Inc.; consultant fees from Kyowa Pharmaceutical Industry Co., Ltd.; Teijin Pharma Ltd.; and Inter Reha Co., Ltd.; and research grants from Teijin Pharma Ltd.

## ETHICS STATEMENT

This study was conducted in accordance with the Declaration of Helsinki.

Approval of the research protocol by an institutional reviewer board: This study was approved by the Ethics Committee of Jikei University (approval no: 30‐154[9175]) on December 13, 2021.

Informed consent: The study objectives and details were posted in an opt‐out document on the website of Jikei University Hospital to fully inform patients about the study details and provide an opportunity to refuse consent to the use of their data.

Registry and the Registration No. of the study/trial: N/A.

Animal Studies: N/A.

## Supporting information


Appendix S1.

## Data Availability

We cannot make the raw data freely available because we did not obtain agreements to release the data from the study participants, and our ethical approval did not include provisions for data release. Researchers interested in collaborative work may contact the corresponding author; however, access to the data requires approval by the institutional ethics committee to maintain the privacy and confidentiality of the participants.
