# Supplementary material for: Effects of repetitive transcranial magnetic stimulation therapy on weight and lipid metabolism in patients with treatment‐resistant depression: A preliminary single‐center retrospective cohort study
Source: Neuropsychopharmacol Rep. 2024 Nov 9;45(1):e12494. doi: 10.1002/npr2.12494 (PMC11660761; doi:10.1002/npr2.12494)
Supplement: Supplementary file 1 — Appendix S1. [file NPR2-45-e12494-s001.docx]

**Supplemental Table 1A. Changes in body weight, BMI, and laboratory data in high BMI group (BMI ≥ 22)**

| **High BMI group (n=28)** | **Change** | | **95% CI** | ***P*** |
| --- | --- | --- | --- | --- |
|  | **Mean** | **SD** |  |  |
| BMI | −0.37 | 0.92 | −0.75 to 0.01 | 0.06 |
| Body Weight (kg) | −0.93 | 2.71 | −2.05 to 0.19 | 0.10 |
| Fasting blood sugar (mg/dl) | −17.67 | 45.06 | −36.70 to 1.36 | 0.07 |
| HbA1c(%) | −0.47 | 1.56 | −1.91 to 0.97 | 0.46 |
| Total cholesterol  (mg/dl) | −17.92 | 37.08 | −33.23 to −2.61 | 0.02 |
| HDL cholesterol  (mg/dl) | −3.44 | 8.84 | −7.09 to 0.21 | 0.06 |
| LDL cholesterol (mg/dl) | −18.39 | 33.95 | −33.07 to −3.71 | 0.02 |
| Triglyceride (mg/dl) | 3.36 | 95.02 | −35.86 to 42.58 | 0.86 |
| Uric acid(mg/dl) | −3.28 | 14.76 | −9.37 to 2.82 | 0.28 |
| AST(IU/L) | −4.80 | 14.66 | −10.85 to 1.25 | 0.12 |
| ALT(IU/L) | −5.32 | 24.53 | −15.44 to 4.80 | 0.29 |
| γ_GTP(IU/L) | −10.84 | 22.46 | −20.11 to −1.57 | 0.02 |
| TSH (µIU/ml) | 0.33 | 0.51 | 0.11 to 0.54 | 0.004 |
| FT3(pg/ml) | −0.19 | 0.47 | −0.39 to 0.01 | 0.06 |
| FT4(ng/dl) | −0.07 | 0.20 | −0.15 to 0.02 | 0.12 |

Abbreviations: 95% CI, 95% confidence interval; BMI, body mass index; ALT, alanine aminotransferase; AST, aspartate aminotransferase; d, Cohen's d; FT3, free triiodothyronine; FT4, free thyroxine; HbA1c, hemoglobin A1c; HDL, high‐density lipoprotein; LDL, low‐density lipoprotein; rTMS, repetitive transcranial magnetic stimulation; SD, standard deviation; TSH, thyroid‐stimulating hormone; γ‐GTP, γ‐glutamyl transpeptidase.

**Supplemental Table 1B. Changes in body weight, BMI, and laboratory data in low BMI group (BMI <22)**

| **low BMI group (n = 10)** | **Change** | | **95% CI** | ***P*** |
| --- | --- | --- | --- | --- |
|  | **Mean** | **SD** |  |  |
| BMI | −0.01 | 0.45 | −0.35 to 0.33 | 0.94 |
| Body Weight (kg) | −0.06 | 1.24 | −1.01 to 0.90 | 0.90 |
| Fasting blood sugar (mg/dl) | −7.00 | 9.90 | −14.61 to 0.61 | 0.07 |
| HbA1c(%) | 0.07 | 0.18 | −0.12 to 0.25 | 0.39 |
| Total cholesterol  (mg/dl) | −6.30 | 20.92 | −21.27 to 8.67 | 0.37 |
| HDL cholesterol  (mg/dl) | −1.90 | 9.84 | −8.94 to 5.14 | 0.56 |
| LDL cholesterol (mg/dl) | −3.70 | 17.70 | −16.36 to 8.96 | 0.53 |
| Triglyceride (mg/dl) | −3.50 | 33.26 | −27.29 to 20.29 | 0.75 |
| Uric acid(mg/dl) | −0.11 | 0.51 | −0.48 to 0.26 | 0.52 |
| AST(IU/L) | 3.20 | 5.57 | −0.79 to 7.19 | 0.10 |
| ALT(IU/L) | 9.30 | 12.84 | 0.11 to 18.49 | 0.05 |
| γ_GTP(IU/L) | 3.70 | 7.72 | −1.82 to 9.22 | 0.16 |
| TSH (µIU/ml) | −0.16 | 0.36 | −0.44 to 0.12 | 0.21 |
| FT3(pg/ml) | −0.25 | 0.15 | −0.37 to −0.13 | 0.001 |
| FT4(ng/dl) | −0.07 | 0.18 | −0.21 to 0.07 | 0.27 |

Abbreviations: 95% CI, 95% confidence interval; BMI, body mass index; ALT, alanine aminotransferase; AST, aspartate aminotransferase; d, Cohen's d; FT3, free triiodothyronine; FT4, free thyroxine; HbA1c, hemoglobin A1c; HDL, high‐density lipoprotein; LDL, low‐density lipoprotein; rTMS, repetitive transcranial magnetic stimulation; SD, standard deviation; TSH, thyroid‐stimulating hormone; γ‐GTP, γ‐glutamyl transpeptidase.
